# A monoclinic modification of 2-[(1,3-benzothia­zol-2-yl)imino­meth­yl]phenol

**DOI:** 10.1107/S1600536810023755

**Published:** 2010-06-26

**Authors:** Abdullah M. Asiri, Salman A. Khan, Kong Wai Tan, Seik Weng Ng

**Affiliations:** a Chemistry Department, Faculty of Science, King Abdul Aziz University, PO Box 80203, Jeddah 21589, Saudi Arabia; b Department of Chemistry, University of Malaya, 50603 Kuala Lumpur, Malaysia

## Abstract

In the title Schiff base, C_14_H_10_N_2_OS, the azomethine double bond is in an *E* configuration; the benzothiazolyl ring (r.m.s. deviation = 0.007 Å) is coplanar with the phenyl­ene ring (r.m.s. deviation = 0.007 Å), the two rings being slightly bent at 2.6 (1)°. The hy­droxy H atom forms an intra­molecular hydrogen bond to the imino group. The bond dimensions of the monoclinic modification are similar to those of the ortho­rhom­bic modification [Liu *et al.* (2009 ▶). *Acta Cryst.* E**65**, o738].

## Related literature

For an ortho­rhom­bic modification of this structure, see: Liu *et al.* (2009 ▶).
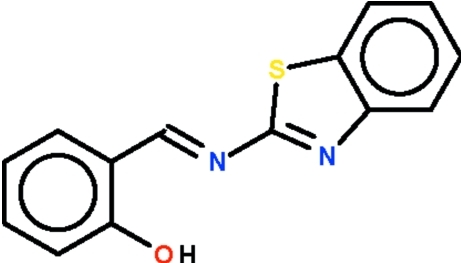


## Experimental

### 

#### Crystal data

C_14_H_10_N_2_OS
*M* *_r_* = 254.30
Monoclinic, 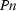

*a* = 8.6391 (4) Å
*b* = 6.2313 (4) Å
*c* = 11.4459 (8) Å
β = 108.893 (1)°
*V* = 582.97 (6) Å^3^
*Z* = 2
Mo *K*α radiation
μ = 0.26 mm^−1^
*T* = 100 K
0.14 × 0.13 × 0.08 mm

#### Data collection

Bruker SMART APEX diffractometer
Absorption correction: multi-scan (*SADABS*; Sheldrick, 1996 ▶) *T*_min_ = 0.964, *T*_max_ = 0.979
5307 measured reflections
2599 independent reflections
2512 reflections with *I* > 2σ(*I*)
*R*_int_ = 0.029

#### Refinement

*R*[*F* ^2^ > 2σ(*F* ^2^)] = 0.040
*wR*(*F* ^2^) = 0.110
*S* = 1.05
2599 reflections
164 parameters
2 restraints
H-atom parameters constrained
Δρ_max_ = 0.40 e Å^−3^
Δρ_min_ = −0.25 e Å^−3^
Absolute structure: Flack (1983 ▶), 1242 Friedel pairs
Flack parameter: 0.27 (8)

### 

Data collection: *APEX2* (Bruker, 2009 ▶); cell refinement: *SAINT* (Bruker, 2009 ▶); data reduction: *SAINT*; program(s) used to solve structure: *SHELXS97* (Sheldrick, 2008 ▶); program(s) used to refine structure: *SHELXL97* (Sheldrick, 2008 ▶); molecular graphics: *X-SEED* (Barbour, 2001 ▶); software used to prepare material for publication: *publCIF* (Westrip, 2010 ▶).

## Supplementary Material

Crystal structure: contains datablocks global, I. DOI: 10.1107/S1600536810023755/nk2043sup1.cif

Structure factors: contains datablocks I. DOI: 10.1107/S1600536810023755/nk2043Isup2.hkl

Additional supplementary materials:  crystallographic information; 3D view; checkCIF report

## Figures and Tables

**Table 1:** Hydrogen-bond geometry (Å, °)

*D*—H⋯*A*	*D*—H	H⋯*A*	*D*⋯*A*	*D*—H⋯*A*
O1—H1⋯N1	0.87	1.73	2.550 (2)	156

**Fig. 1.**
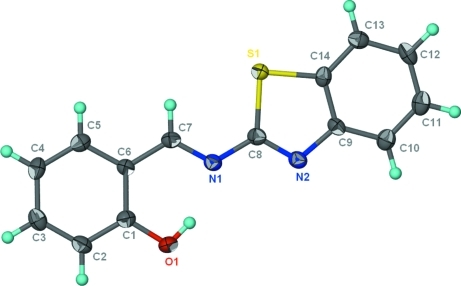
Thermal ellipsoid plot (Barbour, 2001) of C14H10N2OS at the 70% probability level; hydrogen atoms are drawn as spheres of arbitrary radius.

## supplementary crystallographic information

### Comment

The orthorhombic modification of 2-[(1,3-benzothiazol-2-yl)iminomethyl]phenol (Scheme I) is a flat molecule having a (calculated) density of 1.409 g ml^-1^ (Liu *et al.*, 2009). In the monoclinic modification, the packing is more compact (calculated density 1.449 g ml^-1^). The benzisotholyl ring [r.m.s. deviation 0.007 Å] is co-planar with the phenylene ring [r.m.s. deviation 0.007 Å], the two rings being slightly bent by 2.6 (1) ° only. The hydroxy H-atom forms an intramolecular hydrogen bond to the imino group (Fig. 1). The r.m.s. deviation of the non-hydrogen atoms for the least-squares plane is 0.034 Å, and all deviations deviate by 0.002 Å only.

### Experimental

2-Aminobenzothiazole (0.50 g, 4.4 mol) and salicyladehyde (0.66 g, 4.4 mol) were heated in methanol (15 ml) for 5 h. Yellowish-orange crystals deposited when the solution was set aside for a day.

### Refinement

Carbon-bound H-atoms were placed in calculated positions [C–H 0.95 Å, *U*(H) 1.2*U*_eq_(C)] and were included in the refinement in the riding model approximation. The hydroxy H-atom was located in a difference Fourier map, but attempts to refine it even with a distance restraint led to a small temperature factor. The position and temperature factor were not refined. The structure is a racemic twin, the explicit refinement of the Flack parameter from 1242 Friedel pairs gave a value of 0.27 (8).

### Crystal data

**Table:**

C_14_H_10_N_2_OS	*F*(000) = 264
*M**_r_* = 254.30	*D*_x_ = 1.449 Mg m^−^^3^
Monoclinic, *P**n*	Mo *K*α radiation, λ = 0.71073 Å
Hall symbol: P -2yac	Cell parameters from 3896 reflections
*a* = 8.6391 (4) Å	θ = 2.6–28.3°
*b* = 6.2313 (4) Å	µ = 0.26 mm^−^^1^
*c* = 11.4459 (8) Å	*T* = 100 K
β = 108.893 (1)°	Prism, orange
*V* = 582.97 (6) Å^3^	0.14 × 0.13 × 0.08 mm
*Z* = 2	

### Data collection

**Table:**

Bruker SMART APEX diffractometer	2599 independent reflections
Radiation source: fine-focus sealed tube	2512 reflections with *I* > 2σ(*I*)
graphite	*R*_int_ = 0.029
ω scans	θ_max_ = 27.5°, θ_min_ = 2.6°
Absorption correction: multi-scan (*SADABS*; Sheldrick, 1996)	*h* = −11→10
*T*_min_ = 0.964, *T*_max_ = 0.979	*k* = −8→8
5307 measured reflections	*l* = −14→14

### Refinement

**Table:**

Refinement on *F*^2^	Secondary atom site location: difference Fourier map
Least-squares matrix: full	Hydrogen site location: inferred from neighbouring sites
*R*[*F*^2^ > 2σ(*F*^2^)] = 0.040	H-atom parameters constrained
*wR*(*F*^2^) = 0.110	*w* = 1/[σ^2^(*F*_o_^2^) + (0.0831*P*)^2^ + 0.0645*P*] where *P* = (*F*_o_^2^ + 2*F*_c_^2^)/3
*S* = 1.05	(Δ/σ)_max_ = 0.001
2599 reflections	Δρ_max_ = 0.40 e Å^−^^3^
164 parameters	Δρ_min_ = −0.25 e Å^−^^3^
2 restraints	Absolute structure: Flack (1983), 1242 Friedel pairs
Primary atom site location: structure-invariant direct methods	Flack parameter: 0.27 (8)

### Fractional atomic coordinates and isotropic or equivalent isotropic displacement parameters (Å^2^)

**Table:**

	*x*	*y*	*z*	*U*_iso_*/*U*_eq_	
S1	0.50007 (6)	0.06429 (7)	0.49993 (5)	0.01864 (15)	
O1	0.7048 (2)	0.5684 (3)	0.89741 (16)	0.0277 (4)	
H1	0.6924	0.4623	0.8462	0.042*	
N1	0.6095 (2)	0.3254 (3)	0.70823 (16)	0.0186 (4)	
N2	0.7613 (2)	0.0261 (3)	0.69409 (17)	0.0193 (4)	
C1	0.5700 (3)	0.6867 (4)	0.84466 (19)	0.0195 (4)	
C2	0.5445 (3)	0.8696 (4)	0.9059 (2)	0.0222 (4)	
H2	0.6227	0.9096	0.9825	0.027*	
C3	0.4058 (3)	0.9936 (4)	0.8561 (2)	0.0226 (5)	
H3	0.3895	1.1183	0.8985	0.027*	
C4	0.2898 (3)	0.9363 (3)	0.7441 (2)	0.0228 (5)	
H4	0.1937	1.0201	0.7108	0.027*	
C5	0.3153 (3)	0.7571 (4)	0.68168 (19)	0.0214 (4)	
H5	0.2366	0.7193	0.6049	0.026*	
C6	0.4554 (3)	0.6303 (3)	0.72981 (19)	0.0175 (4)	
C7	0.4806 (3)	0.4437 (3)	0.6638 (2)	0.0188 (4)	
H7	0.4019	0.4071	0.5868	0.023*	
C8	0.6349 (3)	0.1448 (3)	0.64566 (19)	0.0184 (4)	
C9	0.7588 (3)	−0.1475 (3)	0.61665 (18)	0.0178 (4)	
C10	0.8777 (3)	−0.3090 (4)	0.6428 (2)	0.0228 (4)	
H10	0.9681	−0.3056	0.7170	0.027*	
C11	0.8612 (3)	−0.4729 (4)	0.5589 (2)	0.0249 (5)	
H11	0.9414	−0.5832	0.5757	0.030*	
C12	0.7290 (3)	−0.4798 (4)	0.4498 (2)	0.0229 (5)	
H12	0.7212	−0.5939	0.3931	0.027*	
C13	0.6081 (3)	−0.3219 (4)	0.4225 (2)	0.0213 (4)	
H13	0.5170	−0.3277	0.3488	0.026*	
C14	0.6257 (3)	−0.1556 (3)	0.5070 (2)	0.0190 (4)	

### Atomic displacement parameters (Å^2^)

**Table:**

	*U*^11^	*U*^22^	*U*^33^	*U*^12^	*U*^13^	*U*^23^
S1	0.0181 (2)	0.0172 (2)	0.0187 (2)	−0.0003 (2)	0.00322 (17)	−0.00262 (19)
O1	0.0216 (9)	0.0314 (10)	0.0232 (9)	0.0063 (7)	−0.0024 (7)	−0.0064 (6)
N1	0.0193 (8)	0.0166 (8)	0.0183 (8)	−0.0016 (7)	0.0040 (7)	−0.0015 (7)
N2	0.0222 (10)	0.0148 (8)	0.0191 (9)	−0.0020 (7)	0.0042 (7)	−0.0008 (7)
C1	0.0181 (10)	0.0220 (10)	0.0181 (9)	−0.0008 (8)	0.0051 (8)	−0.0004 (8)
C2	0.0235 (11)	0.0215 (10)	0.0211 (10)	−0.0032 (9)	0.0066 (9)	−0.0052 (8)
C3	0.0265 (12)	0.0193 (10)	0.0263 (11)	−0.0017 (9)	0.0144 (9)	−0.0023 (8)
C4	0.0228 (12)	0.0211 (11)	0.0255 (11)	0.0054 (8)	0.0095 (9)	0.0051 (8)
C5	0.0197 (10)	0.0245 (10)	0.0190 (9)	0.0002 (9)	0.0048 (8)	0.0025 (8)
C6	0.0179 (10)	0.0178 (9)	0.0164 (9)	−0.0011 (8)	0.0050 (8)	−0.0012 (8)
C7	0.0173 (10)	0.0200 (11)	0.0177 (10)	−0.0029 (8)	0.0035 (8)	−0.0002 (7)
C8	0.0196 (10)	0.0168 (10)	0.0182 (9)	−0.0025 (8)	0.0051 (8)	0.0001 (7)
C9	0.0221 (10)	0.0148 (9)	0.0175 (9)	−0.0025 (8)	0.0077 (8)	−0.0021 (8)
C10	0.0228 (11)	0.0209 (10)	0.0249 (10)	0.0016 (9)	0.0081 (8)	0.0032 (8)
C11	0.0245 (12)	0.0224 (11)	0.0311 (12)	0.0032 (9)	0.0136 (10)	0.0047 (9)
C12	0.0282 (13)	0.0182 (10)	0.0276 (12)	−0.0036 (9)	0.0166 (10)	−0.0058 (9)
C13	0.0205 (10)	0.0224 (11)	0.0208 (10)	−0.0043 (9)	0.0063 (8)	−0.0018 (8)
C14	0.0178 (10)	0.0156 (9)	0.0249 (10)	0.0012 (8)	0.0088 (8)	0.0022 (8)

### Geometric parameters (Å, °)

**Table:**

S1—C14	1.733 (2)	C4—H4	0.9500
S1—C8	1.770 (2)	C5—C6	1.400 (3)
O1—C1	1.345 (3)	C5—H5	0.9500
O1—H1	0.8672	C6—C7	1.442 (3)
N1—C7	1.294 (3)	C7—H7	0.9500
N1—C8	1.389 (3)	C9—C10	1.399 (3)
N2—C8	1.286 (3)	C9—C14	1.402 (3)
N2—C9	1.394 (3)	C10—C11	1.378 (3)
C1—C2	1.393 (3)	C10—H10	0.9500
C1—C6	1.410 (3)	C11—C12	1.393 (4)
C2—C3	1.383 (4)	C11—H11	0.9500
C2—H2	0.9500	C12—C13	1.395 (3)
C3—C4	1.394 (4)	C12—H12	0.9500
C3—H3	0.9500	C13—C14	1.392 (3)
C4—C5	1.381 (3)	C13—H13	0.9500
			
C14—S1—C8	88.21 (11)	N1—C7—H7	119.7
C1—O1—H1	102.4	C6—C7—H7	119.7
C7—N1—C8	121.20 (18)	N2—C8—N1	119.83 (19)
C8—N2—C9	109.65 (18)	N2—C8—S1	116.81 (16)
O1—C1—C2	118.56 (19)	N1—C8—S1	123.36 (17)
O1—C1—C6	121.8 (2)	N2—C9—C10	124.3 (2)
C2—C1—C6	119.7 (2)	N2—C9—C14	115.84 (19)
C3—C2—C1	120.4 (2)	C10—C9—C14	119.9 (2)
C3—C2—H2	119.8	C11—C10—C9	118.8 (2)
C1—C2—H2	119.8	C11—C10—H10	120.6
C2—C3—C4	120.3 (2)	C9—C10—H10	120.6
C2—C3—H3	119.8	C10—C11—C12	121.1 (2)
C4—C3—H3	119.8	C10—C11—H11	119.4
C5—C4—C3	119.7 (2)	C12—C11—H11	119.4
C5—C4—H4	120.2	C13—C12—C11	121.1 (2)
C3—C4—H4	120.2	C13—C12—H12	119.5
C4—C5—C6	121.0 (2)	C11—C12—H12	119.5
C4—C5—H5	119.5	C14—C13—C12	117.7 (2)
C6—C5—H5	119.5	C14—C13—H13	121.2
C5—C6—C1	118.87 (19)	C12—C13—H13	121.2
C5—C6—C7	120.47 (19)	C13—C14—C9	121.5 (2)
C1—C6—C7	120.7 (2)	C13—C14—S1	129.07 (18)
N1—C7—C6	120.7 (2)	C9—C14—S1	109.47 (16)
			
O1—C1—C2—C3	−178.7 (2)	C14—S1—C8—N2	1.30 (18)
C6—C1—C2—C3	1.4 (3)	C14—S1—C8—N1	−178.28 (18)
C1—C2—C3—C4	0.2 (4)	C8—N2—C9—C10	−179.1 (2)
C2—C3—C4—C5	−1.2 (4)	C8—N2—C9—C14	0.2 (3)
C3—C4—C5—C6	0.6 (3)	N2—C9—C10—C11	179.7 (2)
C4—C5—C6—C1	0.9 (3)	C14—C9—C10—C11	0.4 (3)
C4—C5—C6—C7	−180.0 (2)	C9—C10—C11—C12	−0.1 (3)
O1—C1—C6—C5	178.2 (2)	C10—C11—C12—C13	−0.6 (4)
C2—C1—C6—C5	−1.9 (3)	C11—C12—C13—C14	1.1 (3)
O1—C1—C6—C7	−0.9 (3)	C12—C13—C14—C9	−0.8 (3)
C2—C1—C6—C7	179.0 (2)	C12—C13—C14—S1	179.15 (17)
C8—N1—C7—C6	179.8 (2)	N2—C9—C14—C13	−179.33 (19)
C5—C6—C7—N1	−179.6 (2)	C10—C9—C14—C13	0.0 (3)
C1—C6—C7—N1	−0.5 (3)	N2—C9—C14—S1	0.7 (2)
C9—N2—C8—N1	178.51 (18)	C10—C9—C14—S1	−179.89 (17)
C9—N2—C8—S1	−1.1 (2)	C8—S1—C14—C13	179.0 (2)
C7—N1—C8—N2	−177.6 (2)	C8—S1—C14—C9	−1.04 (16)
C7—N1—C8—S1	2.0 (3)		

### Hydrogen-bond geometry (Å, °)

**Table:**

*D*—H···*A*	*D*—H	H···*A*	*D*···*A*	*D*—H···*A*
O1—H1···N1	0.87	1.73	2.550 (2)	156
